# Relationship between Health Decision‐Making Roles and Subjective Memory Ability among Older Adults in Rural Community

**DOI:** 10.1002/alz.088473

**Published:** 2025-01-09

**Authors:** Myonghwa Park, Jinju Kim, Heejung Kim, Eunkyung Seo, Wonmi An

**Affiliations:** ^1^ Chungnam National University, College of Nursing, Daejeon Korea, Republic of (South)

## Abstract

**Background:**

Older adults in rural areas often face the challenge of managing their health decisions due to limited access to medical services. Cognitive function, particularly the awareness and assessment of one’s memory abilities, plays a significant role in the decision‐making process. This study investigates the relationship between subjective memory ability and health‐related decision‐making among older adults in rural areas.

**Methods:**

This research was conducted as a secondary analysis using data from a broader health advancement project in a rural South Korean locality. It examined the roles in health decision‐making, categorizing them into three: active (making decisions independently), shared (making decisions with family members), and passive (having decisions made by family members). Subjective memory ability was assessed on a scale from 0 to 10, reflecting perceived memory ability.

**Results:**

The study involved participants with an average age of 75 years. It found that 47.0% assumed active roles, 48.3% shared roles, and 4.6% passive roles in health decision‐making. Women predominantly assumed active roles (51.7%), while men were more likely to be in shared (50.1%) and passive roles (6.1%). Active roles peaked among those aged 70‐79 (48.1%), shared roles were most common under 70 (50.2%), and passive roles increased after 80 (10.0%). Regarding education, active roles were most frequent among college‐educated individuals, shared roles among middle/high school graduates, and passive roles among those with the least education. The average subjective memory ability scores varied with decision‐making roles: 5.85 for active roles, 5.77 for shared roles, and 4.57 for passive roles. Lower subjective memory ability scores were associated with a higher likelihood of assuming passive roles (*p* = .001).

**Conclusion:**

The study demonstrates that those with lower subjective memory ability scores tend to adopt passive roles, underscoring the influence of perceived autonomy on decision‐making. Therefore, developing support strategies that empower older adults, particularly those with cognitive challenges, to actively participate in their healthcare decisions is essential. Involving families in this process is also important, considering the preferences and cognitive capabilities of older adults.

